# Cytokine Gene Variants as Predisposing Factors for the Development and Progression of Coronary Artery Disease: A Systematic Review

**DOI:** 10.3390/biom14121631

**Published:** 2024-12-19

**Authors:** Fang Li, Yingshuo Zhang, Yichao Wang, Xiaoyan Cai, Xiongwei Fan

**Affiliations:** The Laboratory of Heart Development Research, College of Life Sciences, Hunan Normal University, Changsha 410081, China; 202420142891@hunnu.edu.cn (Y.Z.); 202420142892@hunnu.edu.cn (Y.W.); 202320142782@hunnu.edu.cn (X.C.)

**Keywords:** cytokine variants, pro-inflammatory response, coronary artery disease, meta-analysis, predisposing factors

## Abstract

Coronary artery disease (CAD) is the most prevalent form of cardiovascular disease. A growing body of research shows that interleukins (ILs), such as IL-8, IL-18 and IL-16, elicit pro-inflammatory responses and may play critical roles in the pathologic process of CAD. Single nucleotide polymorphisms (SNPs), capable of generating functional modifications in IL genes, appear to be associated with CAD risk. This study aims to evaluate the associations of ten previously identified SNPs of the three cytokines with susceptibility to or protection of CAD. A systematic review and meta-analysis were conducted using Pubmed, EMBASE, WOS, CENTRAL, CNKI, CBM, Weipu, WANFANG Data and Google Scholar databases for relevant literature published up to September 2024. Odds ratios (ORs) with 95% confidence intervals (CIs) were calculated for the four genetic models of the investigated SNPs in overall and subgroups analyses. Thirty-eight articles from 16 countries involving 14574 cases and 13001 controls were included. The present meta-analysis revealed no significant association between CAD and IL-8-rs2227306 or five IL-16 SNPs (rs8034928, rs3848180, rs1131445, rs4778889 and rs11556218). However, IL-8-rs4073 was significantly associated with an increased risk of CAD across all genetic models. In contrast, three IL-18 (rs187238, rs1946518 and rs1946519) variants containing minor alleles were associated with decreased risks of CAD under all models. Subgroups analyses by ethnicity indicated that IL-8-rs4073 conferred a significantly higher risk of CAD among Asians, including East, South and West Asians (allelic OR = 1.46, homozygous OR = 1.96, heterozygous OR = 1.47, dominant OR = 1.65), while it showed an inversely significant association with CAD risk in Caucasians (homozygous OR = 0.82, dominant OR = 0.85). Additionally, IL-18-rs187238 and IL-18-rs1946518 were significantly associated with reduced CAD risks in East Asians (for rs187238: allelic OR = 0.72, homozygous OR = 0.33, heterozygous OR = 0.73, dominant OR = 0.71; for rs1946518: allelic OR = 0.62, homozygous OR = 0.38, heterozygous OR = 0.49, dominant OR = 0.45). IL-18-rs187238 also demonstrated protective effects in Middle Eastern populations (allelic OR = 0.76, homozygous OR = 0.63, heterozygous OR = 0.72, dominant OR = 0.71). No significant associations were observed in South Asians or Caucasians for these IL-18 SNPs. Consistent with the overall analysis results, subgroups analyses further highlighted a significant association between IL-8-rs4073 and increased risk of acute coronary syndrome (heterozygous OR = 0.72). IL-18-rs187238 was significantly associated with decreased risks of myocardial infarction (MI) (allelic OR = 0.81, homozygous OR = 0.55, dominant OR = 0.80) and multiple vessel stenosis (allelic OR = 0.54, heterozygous OR = 0.45, dominant OR = 0.45). Similarly, IL-18-rs1946518 was significantly associated with reduced MI risk (allelic OR = 0.75, heterozygous OR = 0.68). These findings support the role of cytokine gene IL-8 and IL-18 variants as predisposing factors for the development and progression of CAD.

## 1. Introduction

Coronary artery disease (CAD), characterized by the chronic narrowing of coronary arteries due to atheromatous plaques, can develop myocardial infarction (MI) and the complication of acute coronary syndrome (ACS). Earlier data from Global Burden of Disease reports showed that CAD was the leading cause of death from non-communicable diseases (NCDs) globally, affecting 110 million people and leading to 8.9 million deaths in 2015 [1,2]. Based on the latest reports, CAD remained the leading cause of disability-adjusted life-years (DALYs) for NCDs in 2021, as it had been throughout the previous decade [3]. Increasing evidence has indicated that CAD development and progression are influenced by a series of risk factors besides environmental exposure, including biological heredity and immunological factors. Variations in cytokine genes have garnered particular interest due to their critical role in inflammation, a key underlying mechanism in atherosclerosis and subsequent CAD.

Previous studies initially highlighted the function of interleukin-6 (IL-6) and tumor necrosis factor-alpha (TNF-α) in CAD, followed by a broad focus on other cytokines such as interleukin-8 (IL-8), interleukin-18 (IL-18) and interleukin-16 (IL-16). The protein encoded by the IL-8 gene (also known as CXCL8), located on chromosome 4q13-q21, is recognized for its chemotactic properties, which attract neutrophils to inflammation sites, amplifying the immune response. Since its first description in 1987, numerous in vitro and in vivo assays have linked IL-8 to cardiovascular events, suggesting its potential as a CAD biomarker [4,5]. IL-18, encoded by the gene on chromosome 11q22.2-q22.3, is a pro-inflammatory cytokine that enhances inflammatory responses by inducing interferon-gamma (IFN-γ) production and promoting Th1 cell differentiation. Substantial evidence links IL-18 to atherosclerotic plaque formation and instability, correlating it with MI risk [6,7]. IL-16, located on chromosome 15q26.3, encodes a lymphocyte chemoattractant involved in immune response regulation by affecting the activity of various immune cells. IL-16 has been implicated in numerous inflammatory conditions, including cardiovascular diseases [8,9,10].

Recent studies suggest that single nucleotide polymorphisms (SNPs) of cytokines, as mentioned above, can lead to differential expression levels, potentially modulating the inflammatory response and contributing to CAD pathogenesis. A growing number of studies have explored the association between specific polymorphisms and CAD susceptibility. For instance, the IL-8 polymorphism –251A/T (rs4073) was initially associated with ACS in the Chinese population, but such associations are less evident in Caucasians [11,12]. Similarly, several SNPs within the IL-18 gene have been correlated with CAD, particularly MI, in East Asians but not in other populations [13,14,15,16,17]. A polymorphic variant of IL-16 was first reported to be associated with CAD in the Chinese population [18,19], and its role in ACS has been investigated in Western populations in a recent study [20].

**Figure 1:**
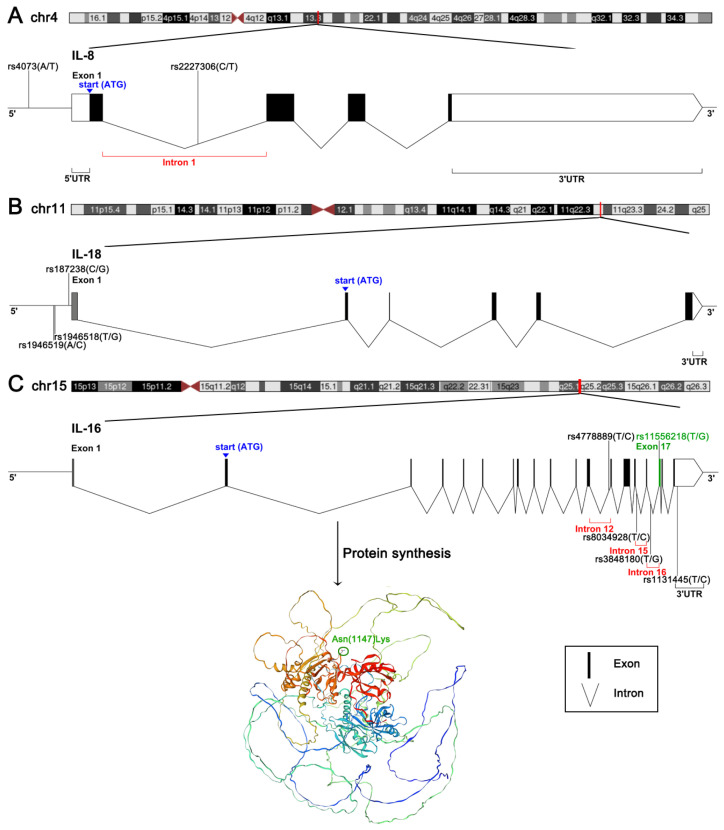
Location of the SNPs on the cytokine genes (**A**) IL-8, (**B**) IL-18 and (**C**) IL-16.

Despite accumulating evidence of the relationship between these cytokine gene SNPs and CAD, conflicting results in the literature necessitate further analysis to provide a more comprehensive overview. In this present study, a systematic meta-analysis was conducted to clarify associations between CAD and ten common polymorphisms of IL-8, IL-18 and IL-16. Figure 1 and Supplementary Table S1 illustrate the locations of nine non-coding SNPs and one missense variant in these cytokine genes. The importance of these cytokines in immune response regulation, along with a bioinformatics analysis predicting the functional effects of the variants (Supplementary Table S1), raises the question: “Are these cytokine gene variations validated to be associated with susceptibility to, or protection against, CAD?”.

## 2. Methods

### 2.1. Literature Search Strategy

The electronic databases Pubmed, EMBASE, Web of Science (WOS), Cochrane Central Register of Controlled Trials (CENTRAL), Chinese National Knowledge Infrastructure (CNKI), Chinese Biomedical Literature Database (CBM), Weipu, WANFANG Data and Google Scholar were used for systematically searching all available literature following the preferred reporting items for systematic reviews and meta-analysis (PRISMA) guidelines (up to September 2024; it has not been registered). The search employed the following items: IL-8/CXCL8, IL-18, IL-16, polymorphism/mutation/variation, myocardial infarction, ischemic heart disease and coronary artery/heart disease, without limits on language. In addition, other original studies of potential relevance were traced back through crossing references in eligible publications.

### 2.2. Selection Criteria

**Figure 2:**
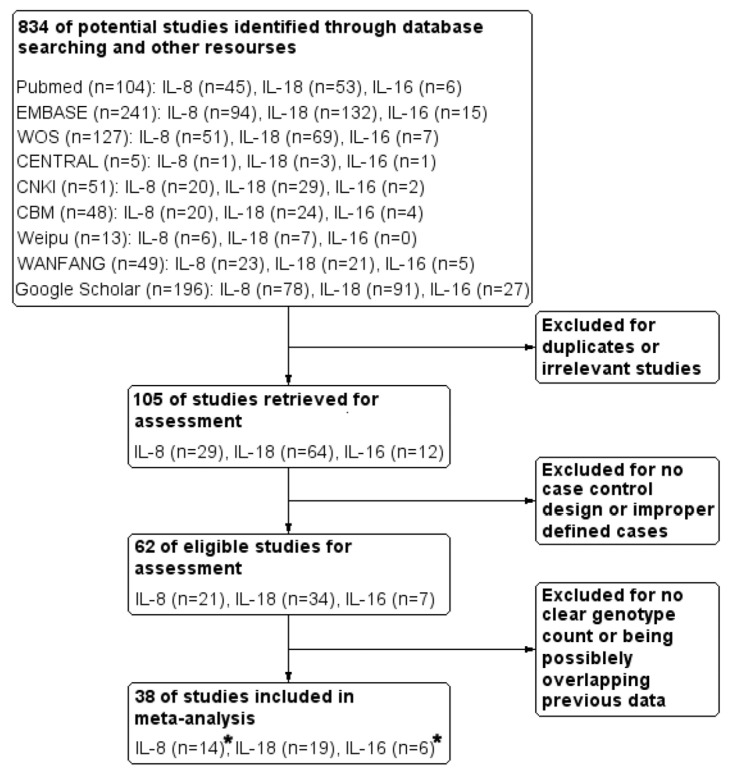
PRISMA flow chart of study inclusion and exclusion. * indicated one article contributed to the meta-analysis of both IL-8 and IL-16.

Eight hundred and thirty-four potential studies were identified by a screen of database searches, followed by full-text retrieval of 105 publications based on the inclusion criteria. Due to ambiguous CAD diagnosis or undesirable experimental design, 43 articles were then excluded. From the remaining 62 publications, 38 were finally included in the meta-analysis after eliminating those with no clear genotype counts or overlapping data. See the PRISMA flow chart in Figure 2. Of all the selected publications, one article contributed to the meta-analysis of both IL-8 and IL-16. Two out of 38 publications, each contained 2 independent cohort studies.

Inclusion criteria: (1) Evaluation of the associations between cytokine gene polymorphisms (rs4073 and rs2227306 of IL-8; rs187238, rs1946518 and rs1946519 of IL-18; rs8034928, rs3848180, rs1131445, rs4778889 and rs11556218 of IL-16) and the risk of CAD. (2) The study should be a case-control design, with clearly verified CAD cases (coronary arteries were angiographically with >50% stenosis). (3) Sufficient data for calculation despite whether the genotype distribution in the control group was in Hardy–Weinberg equilibrium (HWE).

Exclusion criteria: (1) Duplicates, abstracts, reviews, theses, letters or overlapped studies. (2) Case only studies. (3) The study harbored cases with a combination of multiple disease states of cardiovascular events (e.g., congenital cardiac malformation, ischemic cerebral stroke, etc.). (4) Studies with a lack of genotype data.

### 2.3. Data Extraction and Quality Evaluation

Data extraction was carried out independently by two authors from each publication. Information was retrieved as follows: First author name and published year, ethnicity, mean age, gender ratio, sample size, genotyping method, genotype numbers of cases and controls, HWE and quality score. Quality evaluation was performed as previously indicated [21], from which the quality scores comprising the source of cases, source of controls, specimens used for determining genotypes, HWE in controls and total sample size ranged from 0 (worst) to 15 (best). If discrepancies appeared, the third author determined the final assessment.

### 2.4. Statistical Analysis

The chi-square test for HWE was performed to check control quality, and *p* > 0.05 was considered to agree with HWE. Using the software of STATA 12.0 (StataCorp, College Station, TX, USA) and Revman 5.3, calculations of crude odds ratios (ORs) and 95% confidence intervals (CIs) were performed to investigate the associations between 10 variants of interleukins and susceptibility to CAD. Four genetic models, including allelic, homozygous, heterozygous and dominant models, were used to calculate pooled ORs and 95% CIs. I-square (I2) and Z tests were applied, respectively, to determine the heterogeneity and statistical significance of ORs [22]. The fixed-effect model (Mantel–Haenszel method) was used for meta-analysis when no obvious heterogeneity existed within studies (I^2^ < 50%). Otherwise, the random-effect model (DerSimonian and Laird method) was used instead. Stratified analysis was carried out to detect the sources of heterogeneity. Sensitivity analysis was undertaken to evaluate the individual study’s impact on pooled results and the stability of results. Publication bias analysis was performed using Begg’s funnel plot and Egger’s test [23]. It was considered to be statistically significant when *p* < 0.05.

## 3. Results

### 3.1. Characteristics of Eligible Studies

A total of 14 publications [11,12,20,24,25,26,27,28,29,30,31,32,33,34] for IL-8 (15 independent cohorts for rs4073 and 5 independent cohorts for rs2227306), 19 publications [13,14,15,16,17,35,36,37,38,39,40,41,42,43,44,45,46,47,48] for IL-18 (15 independent cohorts for rs187238, 11 independent cohorts for rs1946518 and 3 independent cohorts for rs1946519) and 6 publications [18,19,20,49,50,51] for IL-16 (three independent cohorts for rs8034928, rs3848180 and rs1131445, four independent cohorts for rs4778889 and six independent cohorts for rs11556218) were enrolled in the meta-analysis, including 14,574 cases and 13,001 controls (Figure 2). Among the 38 publications (38 independent cohorts), 12 referred to a group of CAD subtypes involving MI (5 publications), ACS (four publications with five independent cohorts) and single/multiple vessel stenosis (S/MVS, four publications). Asians (East, South and West Asians), Middle Easterners and Caucasians constituted the meta-population. Genotype counts of 10 interleukin SNPs, in terms of both original data and allele frequency calculations in each publication, were accurately checked. The quality scores of individual cohort studies ranged from 7 to 15. For detailed information on included studies, see Table 1.

### 3.2. Results of Meta-Analysis

#### 3.2.1. Associations Between IL-8 SNPs and CAD Risks

**Figure 3:**
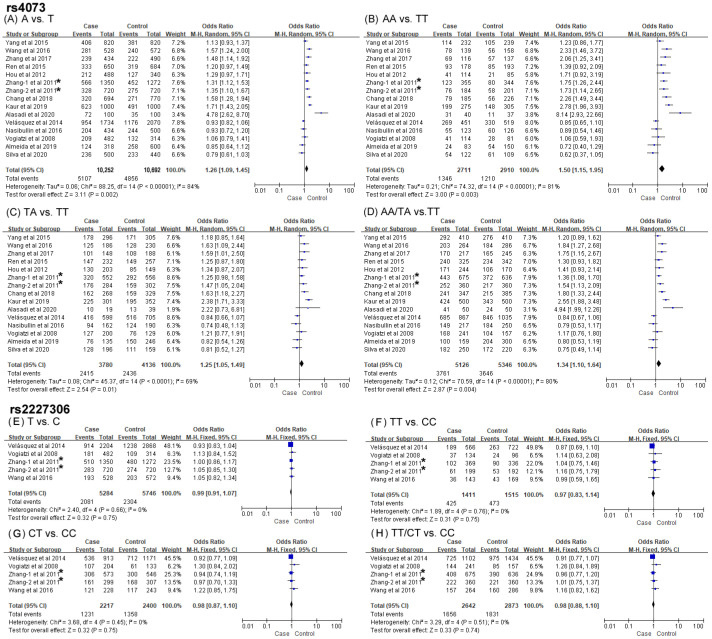
Forest plots of the associations between SNPs rs4073 and rs2227306 of IL-8 and coronary artery disease in the four genetic models [11,12,20,24,25,26,27,28,29,30,31,32,33,34]. (**A**) A vs. T, (**B**) AA vs. TT, (**C**) TA vs. TT, (**D**) AA/TA vs. TT, (**E**) T vs. C, (**F**) TT vs. CC, (**G**) CT vs. CC, (**H**) TT/CT vs. CC. * indicated two independent cohorts for investigation of rs4073 and rs2227306 polymorphisms included in one publication.

Associations of *IL-8* gene variations (rs4073 and rs2227306) with the risk of CAD are displayed in Figure 3 and Table 2. The pooled meta-analysis for correlations between the two SNPs and CAD risks recruited 5126 cases and 5346 controls and 2642 cases and 2873 controls, respectively.

Significant increases in CAD risks were detected to be associated with rs4073 in the four genetic models: the allelic (OR = 1.26, 95% CI = 1.09–1.45, *p* = 0.002), homozygous (OR = 1.50, 95% CI = 1.15–1.95, *p* = 0.003), heterozygous (OR = 1.25, 95% CI = 1.05–1.49, *p* = 0.01) and dominant models (OR = 1.34, 95% CI = 1.10–1.64, *p* = 0.003) (Figure 3A–D). Subgroups analyses by ethnicity, sample size and quality score indicated significant associations between rs4073 and CAD risks in populations of Asians (incl. both Chinese and non-Chinese), the small-sized group and the high-quality group under all genetic models, which was in line with the overall analysis. No associations were found between the large-sized group and the low-quality group. Interestingly, significantly decreased risks of CAD were observed in Caucasians under the homozygous and dominant models, accompanied by a slight weakening significance in allelic contrast and heterozygous model. Additional subgroups analyses stratified by clinical manifestation suggested that distributions of the heterozygous model of rs4073 were significantly increased in the ACS group. However, no obvious associations were found in the non-ACS group (Table 2).

Neither the overall nor CAD subgroup showed an apparent association between rs2227306 and CAD susceptibility using the ACS analysis (Figure 3E–H and Table 2).

#### 3.2.2. Associations Between IL-18 SNPs and CAD Risks

**Figure 4:**
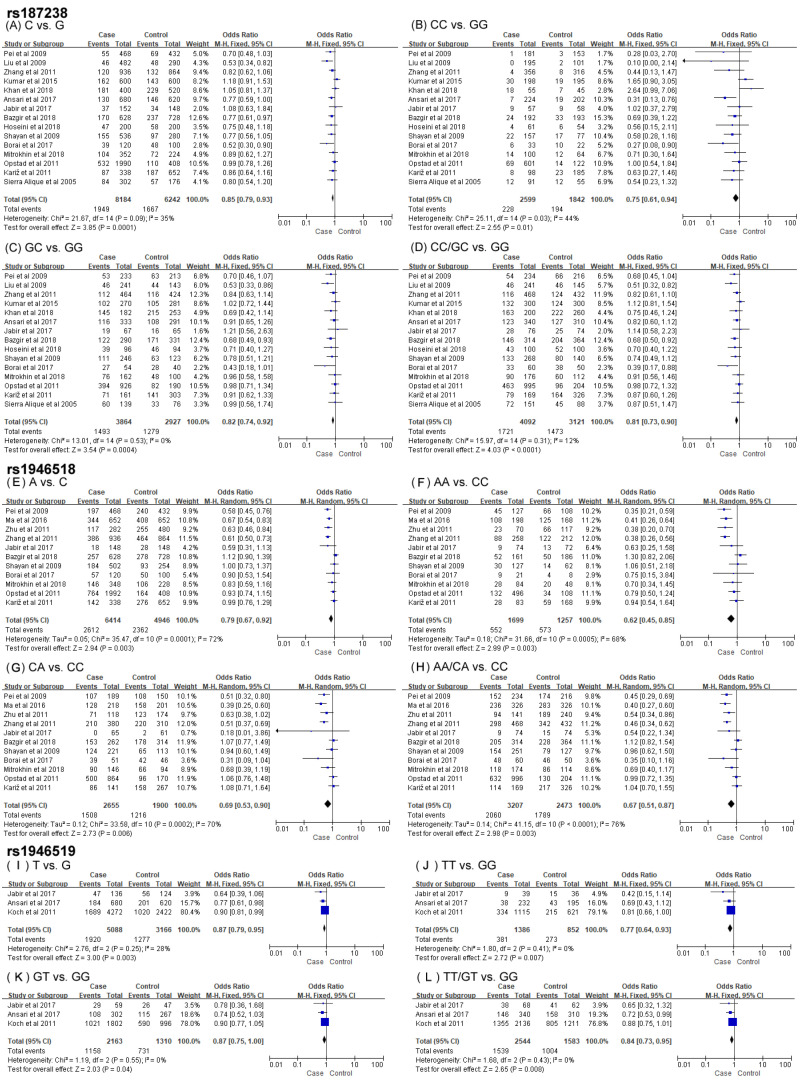
Forest plots of the association between SNPs rs187238, rs1946518 and rs1946519 of IL-18 and coronary artery disease in the four genetic models [13,14,15,16,17,35,36,37,38,39,40,41,42,43,44,45,47,48]. (**A**) C vs. G, (**B**) CC vs. GG, (**C**) GC vs. GG, (**D**) CC/GC vs. GG, (**E**) A vs. C, (**F**) AA vs. CC, (**G**) CA vs. CC, (**H**) AA/CA vs. CC., (**I**) T vs. G, (**J**) TT vs. GG, (**K**) GT vs. GG, (**L**) TT/GT vs. GG.

It can be seen from Figure 4 and Table 3 that participants in the pooled meta-analysis for associations of *IL-18* rs187238, rs1946518 and rs1946519 with CAD risks had 4092 cases and 3121 controls, 3207 cases and 2473 controls and 2544 cases and 1583 controls, respectively.

Significant associations between rs187238 and decreased risk of CAD were investigated in the allelic contrast (OR = 0.85, 95% CI = 0.79–0.93, *p* < 0.001), homozygous model (OR = 0.75, 95% CI = 0.61–0.94, *p* = 0.01), heterozygous model (OR = 0.82, 95% CI = 0.74–0.92, *p* < 0.001) and dominant model (OR = 0.81, 95% CI = 0.73–0.90, *p* < 0.001) (Figure 4A–D). Following subgroups analyses by ethnicity, sample size and quality score, the same significant associations were detected in East Asians, Middle Easterners, the small-sized group and the high-quality group. The stratified results demonstrated a lack of association between rs187238 and susceptibility to CAD in South Asians and Caucasians groups, the large-sized group and the low-quality group. Further subgroups analyses based on clinical manifestation indicated significant relationships between allelic, homozygous and dominant models of rs187238 and CAD risks in the MI group. A significant tendency to an association between rs187238 and CAD was found in the MVS group in contrast to that in the SVS group under the four genetic models (Table 3).

The pooled data for allelic contrast (OR = 0.79, 95% CI = 0.67–0.92, *p* = 0.003), homozygous model (OR = 0.62, 95% CI = 0.45–0.85, *p* = 0.003), heterozygous model (OR = 0.69, 95% CI = 0.53–0.90, *p* = 0.006) and dominant model (OR = 0.67, 95% CI = 0.51–0.87, *p* = 0.003) (Figure 4E–H) of rs1946518 exhibited significant associations with a decreased risk of CAD. The significance was markedly strengthened in East Asians but disappeared in Middle Easterners and Caucasians during subgroups analyses by ethnicity. Stratified data by sample size and quality score revealed consistent significant correlations between rs1946518 and CAD in the small-sized group and the high-quality group under the four genetic models. Such associations were also observed in the low-quality group under the heterozygous and dominant models. Albeit nonsignificant, a subgroup analysis by clinical manifestation uncovered associations between rs1946518 and CAD in the MI group among all genetic models (Table 3).

In the light of the pooled information, the polymorphism rs1946519 was critically associated with a decreased risk of CAD in the allelic contrast (OR = 0.87, 95% CI = 0.79–0.95, *p* = 0.003), homozygous model (OR = 0.77, 95% CI = 0.64–0.93, *p* = 0.007), heterozygous model (OR = 0.87, 95% CI = 0.75–1.00, *p* = 0.04) and dominant model (OR = 0.84, 95% CI = 0.73–0.95, *p* = 0.008) (Figure 4I–L).

#### 3.2.3. Associations Between IL-16 SNPs and CAD Risks

**Figure 5:**
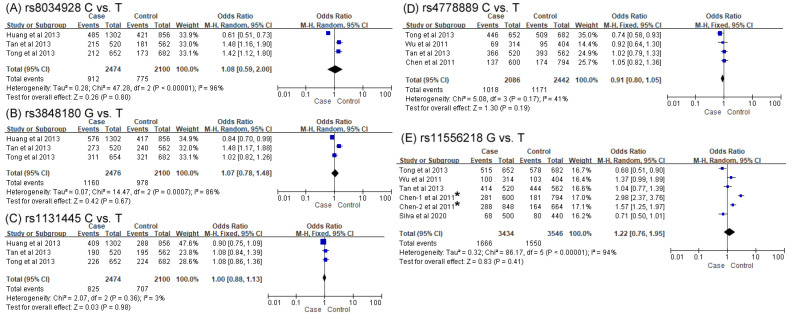
Forest plots of the association between SNPs rs8034928, rs3848180, rs1131445, rs4778889 and rs11556218 of IL-16 and coronary artery disease in the allelic model [18,19,20,49,50,51]. (**A**) C vs. T, (**B**) G vs. T, (**C**) C vs. T, (**D**) C vs. T and (**E**) G vs. T. * indicates two independent cohorts for investigation of rs11556218 polymorphism included in one publication.

The results of the meta-analysis for the associations of five *IL-16* SNPs with CAD risks are shown in Figure 5 and Table 4. Accordingly, 1237 cases and 1050 controls were included in the meta-analysis to examine the effect of rs8034928, rs3848180 and rs1131445 on CAD risks. With regard to investigations of correlations between rs4778889 and rs11556218 and the risk of CAD, 1043 cases and 1221 controls and 1717 cases and 1773 controls were enrolled, respectively.

For the overall analysis of the five SNPs, none exhibited associations of individual variants with CAD susceptibility in any genetic model (Figure 5 and Table 4, only allelic models shown in Figure 5). Furthermore, no association between rs4778889 or rs11556218 and CAD risk was found when stratified by quality score.

### 3.3. Exploration of Heterogeneity

In view of the fact that heterogeneity was present in the studies, its sources were explored thereafter. Significant heterogeneity (*p* < 0.001, I^2^ ranged from 69 to 84%; Figure 3) was observed in the four genetic models during the overall analysis of associations of *IL-8* rs4073 with CAD. Subgroups analyses suggested that ethnicity largely accounted for the heterogeneity among studies (Table 2). As the association results remained significant, reduced heterogeneity was found between all models of this polymorphism and CAD in the Chinese group (*p* > 0.05, I^2^ ranged from 0 to 20%). Moreover, the high heterogeneity existing in allelic, homozygous and dominant (I^2^ ranged from 54 to 73%) models of rs4073 and CAD in the ACS group (five cohorts) was mainly ascribed to the two Western studies. Meta-analysis with the exclusion of the Caucasian populations led to a substantial decrease in heterogeneity (*p* > 0.05, I^2^ ranged from 0 to 9%) and further detection of significant associations (allelic: *p* < 0.0001; homozygous: *p* < 0.0001; dominant: *p* = 0.0001) between rs4073 and an increased risk of ACS in three models. For the analysis of the relationship between *IL-8* rs2227306 and the risk of CAD, no heterogeneity was observed in any studies (Table 2).

There was no heterogeneity displayed among the overall analyses of associations between rs187238 and rs1946519 of IL-18 and susceptibility to CAD (Table 3), while significant heterogeneity (*p* < 0.001, I^2^ ranged from 68 to 76%; Figure 4) was detected in the four genetic models of *IL-18* rs1946518. Ethnicity similarly contributed to this heterogeneity to a large extent since the significant heterogeneity disappeared among all models (*p* > 0.05, I^2^ = 0) in the Chinese group, accompanied by unchanged significant associations between rs1946518 and CAD (Table 3). Not surprisingly, significant heterogeneity (*p* < 0.05, I^2^ ranged from 70 to 79%) observed in the four models of rs1946518 in the MI group (4 studies) also disappeared in further subgroups analyses involving Chinese populations only (2 studies; *p* > 0.05, I^2^ = 0). This stratified analysis illustrated a more significant association of rs1946518 with a decreased risk of MI (for all models: *p* < 0.0001).

The great heterogeneity (*p* < 0.001, I^2^ ranged from 84 to 96%) detected in the four models of *IL-16* rs8034928 was ascribable to the inclusion of Huang et al.’s study because the heterogeneity dropped to 0% when this particular study was removed. However, the cause of the large heterogeneity observed in the allelic (I^2^ = 86%), homozygous (I^2^ = 83%) and dominant models (I^2^ = 75%) of *IL-16* rs3848180 could not be identified. Significant heterogeneity (*p* < 0.001, I^2^ ranged from 88 to 96%) was found in the overall analysis of the association between *IL-16* rs11556218 and CAD. Subgroups analyses indicated that significant heterogeneity was retained among the four models in the high-scoring group (Table 4). This high heterogeneity largely decreased in the homozygous (*p* = 0.61, I^2^ = 0%) model in the low-quality group. No heterogeneity was found in all models of rs1131445 and rs4778889.

### 3.4. Sensitivity Analysis and Publication Bias

Considering the limited number of studies included and the high heterogeneity that existed in partial meta-analysis, the following analyses were focused on studies recruiting more than five cohorts.

**Figure 6:**
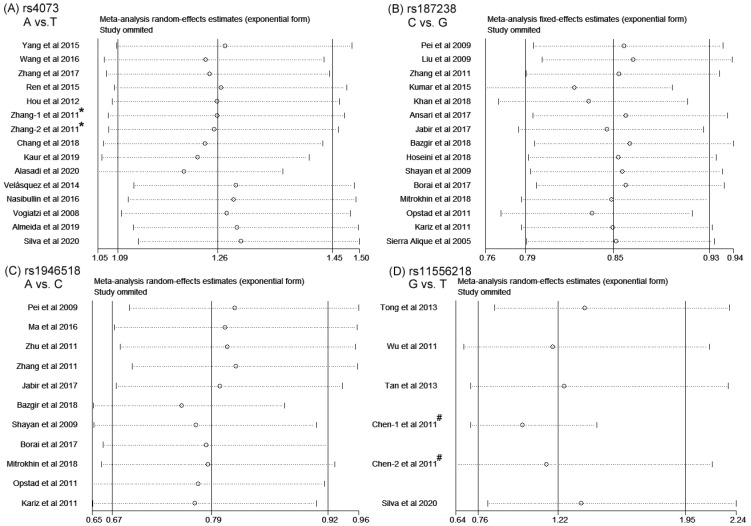
The influence of each study by the removal of individual studies for SNPs rs4073 of IL-8, rs187238 and rs1946518 of IL-18 and rs11556218 of IL-16 and coronary artery disease in the allelic model [11,12,13,14,15,16,17,18,19,20,24,25,26,27,28,29,30,31,32,33,34,35,36,37,38,39,40,41,43,44,45,47,48,50,51]. (**A**) A vs. T, (**B**) C vs. G, (**C**) A vs. C, (**D**) G vs. T. * indicated two independent cohorts for investigation of rs4073 polymorphism included in one publication. ^#^ indicated two independent cohorts for investigation of rs11556218 polymorphism included in one publication.

A sensitivity analysis was conducted to examine whether individual studies influence pooled results, omitting one study at a time. The pooled ORs and 95% CIs of the correlation between *IL-8* rs4073, *IL-18* rs187238, *IL-18* rs1946518 and *IL-16* rs11556218 and CAD were not significantly altered in all genetic models (allelic models are shown in Figure 6).

**Figure 7:**
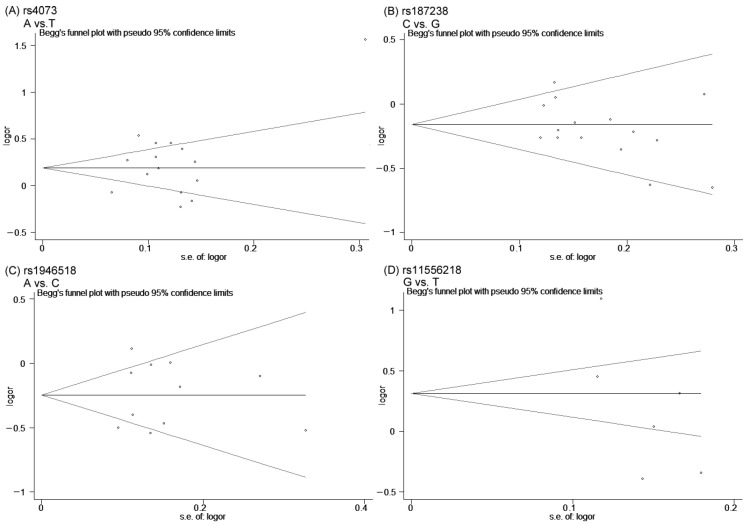
Begg’s funnel plot analysis for publication bias between SNPs rs4073 of IL-8, rs187238 and rs1946518 of IL-18 and rs11556218 of IL-16 and coronary artery disease risk in allelic model. (**A**) A vs. T, (**B**) C vs. G, (**C**) A vs. C, (**D**) G vs. T.

Next, Egger’s linear regression test was performed to evaluate the publication bias. As indicated in Table 5, the *p*-values of Egger’s tests for four SNPs (rs4073, rs187238, rs1946518 and rs11556218) were greater than 0.05 in all analyses. Furthermore, the symmetrical shapes of Begg’s funnel plots (Figure 7, allelic models are shown) concurred with the quantitative assessments of Egger’s tests. These results demonstrated that obvious publication bias did not exist in the meta-analysis.

## 4. Discussion

In the current meta-analysis, pooled results suggested significant associations of four cytokine gene variations (*IL-8* rs4073, *IL-18* rs187238, *IL-18* rs1946518 and *IL-18* rs1946519) with susceptibility to CAD. The A allele, homozygous AA genotype or heterozygous TA genotype of rs4073 were associated with increased risks of CAD. In contrast, individuals who had the C allele, CC genotype or GC genotype (rs187238) or those with the A allele, AA genotype or CA genotype (rs1946518) or those with the T allele, TT genotype or GT genotype (rs1946519) were at lower risk of CAD. No evidence in the present analysis supported the correlation between *IL-8* rs2227306 or five *IL-16* SNPs (rs8034928, rs3848180, rs1131445, rs4778889 and rs11556218) and CAD risks.

In the past two decades, large-scale genome-wide association studies (GWAS) implicating different populations have progressively identified a few hundred CAD susceptibility loci to clarify the genetic architecture of this complex disease [52]. Despite the lack of corresponding loci in the GWAS screen, numerous investigations have examined the association between SNPs in interleukins (e.g., IL-8, IL-18 and IL-16) and CAD risk. However, inconsistent results in these studies may be ascribed to factors such as gender imbalances, small sample size, methodological differences in genotyping and variations in diagnostic criteria. To address these inconsistencies, we, and others, performed meta-analyses based on the case-control studies to provide a more comprehensive and precise estimation of the association between selective interleukin SNPs and CAD risk. Discordant results were found for CAD susceptibility related to the appearance of the allele/genotype of interleukin variants for the meta-analysis below. Whereas Zhang et al. revealed an association between IL-8 rs4073 AA + TA genotype (dominant model) and increased risk of CAD [53], Wu et al. observed an additional association of A allele/AA genotype (allelic model and homozygous model) with higher CAD risk [54]. Concerning IL-18 polymorphisms, two studies conducted by Yang et al. and Zheng et al. associated the appearance of the mutant allele of rs187238 and rs1946518 with higher risks of CAD [55,56], while Lian et al. showed a significantly reduced risk of CAD was correlated with rs187238 in the heterozygous model and dominant model [57]. Compared to these data, our findings demonstrated a significant association of rs4073/rs187238/rsrs1946518 with CAD susceptibility under all genetic models. It is likely attributable to approximately 3~6 more literature enrollments, which incorporated extra participants, up to three thousand, as well as more appropriate publication selection (e.g., excluding studies containing cardiovascular disease, overlapped cohorts, etc.) in our analysis. Following subgroups analyses by ethnicity, our observation of the association between rs4073 and increased CAD risk in East Asians (i.e., the Chinese population) concurred with those seen in other studies [54,58]. Asian cohort studies in our analyses implied that such an association also occurred in South Asians and West Asians. Differing from their studies, it is worthwhile to note that in the present analysis, the rs4073 A allele/AA genotype carriers had a significantly decreased risk for CAD in Caucasians. Consistent with the earlier research, our stratified analysis indicated a decreased CAD risk with rs187238 in East Asians [57]. Unexpectedly, the protective effect conferred by this SNP for CAD was also found in Middle Eastern populations but not in South Asians or Caucasians. Similarly, we detected an association between rs1946518 and a reduced risk of CAD in East Asians. No association between such SNP and CAD, however, was found in South Asians or Caucasians. Conflicting findings of CAD susceptibility with these variants in different ethnic groups globally might be tightly connected with individual genetic background, epigenetic events or environmental impacts or synthetic actions of these factors.

It has been proposed that pro-inflammatory cytokines, including IL-8 and IL-18, play crucial roles in the initiation, progression and complications of atherosclerosis (AS) [59,60]. These interleukins are thought to mediate atherosclerotic plaque destabilization and increase plaque vulnerability, hence resulting in ACS [61,62]. A series of studies have suggested a detrimental role of the inflammasomes in the progression of AS and MI, with IL-18 identified as a downstream effector in the correlated inflammatory pathway [63,64]. Several lines of evidence have shown that inflammasome polymorphisms are linked to the development of ACS and increased production of IL-18 [63,65,66,67]. Clinical and experimental data suggest a proatherogenic effect of IL-18 [68], while IL-16 may exert a protective effect against AS [69]. Furthermore, mounting evidence supports the idea that circulating cytokines serve as inflammatory biomarkers in the development of CAD and correlate with its severity [5]. Patients with ACS/acute myocardial infarction (AMI) had higher plasma IL-8 levels than healthy subjects in Canadian [70] and Japanese populations [71] or CAD-free controls in the Chinese population [72]. Similarly, serum IL-18 concentrations were significantly increased in ACS/AMI patients as compared to healthy volunteers in Chinese [73,74,75,76], Swedish [77], Egyptian [78], Greece [79] and Indonesia populations [80] or CAD-free controls in French [81] and Chinese populations [82]. These findings align with those of a recent meta-analysis [83,84]. Sun et al. also observed increased IL-18 concentrations in patients with S/MVS as compared to healthy controls [75]. Additional studies from an Isreal cohort and a Chinese cohort reported elevated IL-18 levels in patients with stable angina pectoris (SAP) and unstable angina pectoris (UAP) than in healthy [85] or CAD-free controls [82]. A recent retrospective study in a German population found higher serum levels of IL-16 in patients with and without ST-segment elevation myocardial infarction compared with CAD-free controls [86]. However, contradictory findings have been observed regarding pro-inflammatory cytokine measurement in different CAD manifestations. Circulating levels of IL-8 were significantly higher in patients with ACS/MI than those with SAP in German [87] and Chinese populations [72], while no difference was found between ACS/AMI and SAP patients in Greece [88] and Norwegian populations [89] or between ACS/AMI and UAP patients in a Dutch population [90]. A very recent study using CAREBANK and FACT cohorts reported elevated levels of IL-8 in ACS patients compared to stable CAD patients [91]. According to reams of research, ACS patients exhibited significantly higher plasma levels of IL-18 in comparison with SAP in the Chinese population [73,74,75,82] and UAP in the Japanese population [92]. Conversely, Mallat and colleagues detected higher serum IL-18 levels in patients with MI and UAP than those with SAP [81]. The divergent results across studies may be partially explained by the relatively small sample sizes, patient histories regarding coronary intervention and medications, differences in control subject definitions and variations in blood sample collection and processing methods. Nonetheless, it is noteworthy that both IL-8 [93,94] and IL-18 [73,95,96,97] have been suggested as independent predictors of future adverse cardiovascular events in patients with ACS. Furthermore, the balance between pro-inflammatory and anti-inflammatory cytokines (e.g., the ratio of IL-8/IL-10 or IL-18/IL-10) may act as predictors of recurrent coronary outcomes in patients with SAP [98] or ACS [73,99,100].

Delineation of underlying genomic and genetic factors underlying specific diseases aids in advancing diagnostic and therapeutic approaches. Genetic variants are hypothesized to contribute to the regulatory function, gene expression and resultant clinical traits or phenotypes by influencing mRNA transcription, processing and translation. Various bioinformatics tools were utilized to predict the functional effects of ten variants analyzed in this study (see Supplementary Materials). The regulatory potential of nine non-coding polymorphisms—located within the 5’UTR, intronic and 3’UTR regions—and one non-synonymous coding variant was evaluated based on their co-localization with functional regulatory loci. Supported by predictive functional annotations, the missense variant was suggested as a CAD-causing candidate. Although the exact mechanisms remain to be elucidated, several factors provide insight into the potential impacts of SNPs on disease via translational regulation: (1) Variations or epitranscriptomic modifications within 5’UTR may affect protein synthesis initiation [101]. (2) SNPs in intronic or 3’UTR regions may interrupt interactions between RNA and RNA-binding proteins or microRNAs, thereby altering translation efficiency [102]. (3) Genetic variants can cause ribosome occupancy changes and consequently modify the translation elongation rate [103].

Clinical investigations and experimental follow-ups indicate that gene expression and serum levels of cytokines can be affected by several factors, including genetic constitution. A set of SNPs within the promoter, intron and exon regions of IL8/IL-18 has been shown to influence the gene transcription and circulating levels [12,104,105,106,107,108,109]. The haplotypes, also harboring polymorphisms that were investigated in our study, have been found to be associated with the expression or plasma concentrations of these cytokines [106,109,110,111]. Genetic loci of IL-18 and IL-16 have been identified to be associated with circulating levels of two cytokines in a GWAS [65]. Collectively, genetic variations additionally contribute to the development of CAD. An earlier study reported that the combined genotype AA^rs4073^TT^rs2227306^ of IL-8 was independently associated with a reduced risk of ACS in the Greek population [11]. Remarkably, the single genotype TA^rs4073^ was observed to be correlated with increased ACS risk in our stratified meta-analysis. Although an association between rs4073 AA genotype and decreased risk of CAD was observed in Caucasians in the current meta-analysis, larger western populations need to be recruited to testify to the possible protective effect of IL-8. A global gene-wide test involving American participants showed an association between an IL-18 haplotype possessing the rs1946519 G allele and a lower MI risk [112]. On the contrary, Koch et al., utilizing a German cohort, found that another haplotype containing the complementary rs1946519 T allele was correlated with a decreased risk of MI [42]. In combination with the latter large-scale case-control study, our meta-analysis confirmed that specific T allele carriers of rs1946519 had a strongly decreased risk of CAD. Haplotypic analysis concerning seven tag SNPs (three SNPs of TNF-α, three SNPs of IL-1β and IL-18 rs187238) in an Indian cohort revealed a profound association of related haplotypes with CAD risk [41]. The present subgroups analyses further demonstrated a significant association or inclination of IL-18 polymorphisms (rs187238 C allele or rs1946518 A allele) and a reduced risk of MI, supporting the protective role of IL-18 variants containing minor alleles in CAD development and progression. The haplotypes of IL-16 (rs8034928-rs3848180-rs4577037-rs1131445) in the Chinese population appeared to be related to the risk of CAD [49]. Our computation-based functional effect analysis predicted a potential causal association between IL-16 rs11556218 and CAD risk. In line with this prediction, Yuan et al. discovered a high correlation between the rs11556218 T allele and rs4778636 G allele and suggested an association of IL-16 with CAD through Mendelian randomization analysis [10]. Nonetheless, the individual variant referred to exacerbation or protection of CAD and awaits further study as none of the single investigated IL-16 SNPs was found to be associated with CAD risk in our meta-analysis.

This study’s limitations primarily stem from reproducibility challenges, which are influenced by factors such as design, sample size, clinical characteristics, gene–gene interregulations, gene–environmental interactions and genetic and environmental heterogeneity across global populations. In the present meta-analysis, the cohorts observed mainly came from populations in China (seven studies—50.0% for IL-8, five studies—27.8% for IL-18 and five studies—83.3% for IL-16). Although the reviewed publications provided a broader range of populations and examined the associations of four SNPs in two cytokine genes with CAD risk, the meta-analysis did not yield sufficient evidence to establish associations for *IL-8* rs2227306 and *IL-16* SNPs. Stratified analyses of *IL-8* rs4073, *IL-18* rs187238 and *IL-18* rs1946518 with CAD risk revealed an ethnic bias, contributing to the observed heterogeneity. The apparent heterogeneity in generalized analyses of rs4073 and rs1946518 with CAD susceptibility was principally attributable to ethnicity. Reduced heterogeneity and concurrent notable associations between these polymorphisms and risks of ACS and MI detected in the subgroups analyses appeared to be dependent on the inclusion of the Chinese population. Environmental variations among populations affect disease outcomes by altering individual risk factors and immune responses, thereby impacting result consistency. Despite these limitations, our study provided significant contributions to addressing the main research issue and demonstrated standardization and validation, facilitating an efficient qualitative synthesis of the data.

## 5. Conclusions

The findings generated in this meta-analysis highlighted the significance of pro-inflammatory cytokines as susceptibility loci for CAD. Significant associations were observed between four interleukin intergenic SNPs and CAD risk. However, conflicting results from subgroups analyses underscore the need for immunogenetic studies in large and more diverse cohorts. In addition, stratified analyses by clinical manifestation suggested that cytokine gene variants may predispose individuals to the development and progression of CAD. Further research is warranted to elucidate the pathophysiological mechanisms underlying these associations, thereby addressing gaps in the understanding of immunogenetic influences on outcomes of CAD. Taken together, our findings reinforce prior evidence of the correlation between IL-8/IL-18 polymorphisms and CAD risk while observing the lack of association for IL-16 SNPs.

## Figures and Tables

**Table 1:** Characteristics of included studies on cytokine genes genotype [11,12,13,14,15,16,17,20,24,25,26,27,28,29,30,31,32,33,34,35,36,37,38,39,40,41,42,43,44,45,46,47,48].

Author, Year	Ethnicity	Mean Age Case/Control	Gender (%Male) Case/Control	Sample Size Case/Control	Genotyping Method	Genotype						HWE	Quality Score ^§^
						Case			Control				
	East Asian					IL-8 rs4073							
						AA	AT	TT	AA	AT	TT		
Yang et al., 2015	Chinese	NA	57.8/43.2	410/410	PCRRFLP	114	178	118	105	171	134	0.001	9
Wang et al., 2016	Chinese	NA	63.6/54.5	264/286	PCR-RFLP	78	125	61	56	128	102	0.170	12
Zhang et al., 2017	Chinese	NA	65.9/55.9	217/245	PCR-RFLP	69	101	47	57	108	80	0.084	11
Ren et al., 2015	Chinese	NA	59.7/56.7	325/342	MassARRAY	93	147	85	85	149	108	0.021	9
Hou et al., 2012 **^ACS^**	Chinese	64.1 ± 8.5/63.6 ± 8.6	63.9/54.1	244/170	PCR-RFLP	41	130	73	21	85	64	0.373	11
Zhang-1 et al., 2011 *^,ACS^	Chinese	56.4 ± 9.9/55.8 ± 10.4	55.1/55.7	675/636	PCR-RFLP	123	320	232	80	292	264	0.957	13
Zhang-2 et al., 2011 *^,ACS^	Chinese	55.5 ± 10.3/54.7 ± 10.4	55.6/55.6	360/360	PCR-RFLP	76	176	108	58	159	143	0.221	12
Chang et al., 2018	Chinese	60.5 ± 9.8/59.6 ± 9.2	61.9/63.6	347/385	PCR-RFLP	79	162	106	56	159	170	0.063	12
	**South Asian**												
Kaur et al., 2019	Indian	56.1 ± 9.6/51.0 ± 10.2	74.0/79.4	500/500	TP-ARMS-PCR	199	225	76	148	195	157	<0.001	11
	**West Asian**												
Alasadi et al., 2020	Iraqis	NA	70.0/74.0	50/50	TP-ARMS-PCR	31	10	9	11	13	26	0.002	7
	**Caucasian**												
Velásquez et al., 2014 **^MI^**	Swedish	NA	70.2/67.5	867/1035	TaqMan	269	416	182	330	516	189	0.608	15
Nasibullin et al., 2016	Tatar	53.6 ± 5.8/50.5 ± 6.0 NA	NA	217/250	Site-specific PCR	55	94	68	60	124	66	0.907	11
Vogiatzi et al., 2008 **^ACS^**	Greek	64.0 ± 0.7/63.0 ± 1.0	81.7/71.3	241/157	PCR-RFLP	41	127	73	28	76	53	0.933	10
Almeida et al., 2019	Brazilian	60.4/40.3	68.5/50.0	159/300	PCR-RFLP	24	76	59	54	150	96	0.729	11
Silva et al., 2020 **^ACS^**	Brazilian	NA/48.0 ± 6.3	NA/875.4	250/220	PCR-RFLP	54	128	68	61	111	48	0.851	11
						***IL-8*** **rs2227306**							
						**CC**	**CT**	**TT**	**CC**	**CT**	**TT**		
Zhang-1 et al., 2011 *^,ACS^	Chinese	56.4 ± 9.9/55.8 ± 10.4	55.1/55.7	675/636	PCR-RFLP	267	306	102	246	300	90	0.924	13
Zhang-2 et al., 2011 *^,ACS^	Chinese	55.5 ± 10.3/54.7 ± 10.4	55.6/55.6	360/360	PCR-RFLP	138	161	61	139	168	53	0.847	12
Velásquez et al., 2014 **^MI^**	Swedish	NA	70.2/67.5	1102/1434	TaqMan	377	536	189	459	712	263	0.651	15
Vogiatzi et al., 2008 **^ACS^**	Greek	64.0 ± 0.7/63.0 ± 1.0	81.7/71.3	241/157	PCR-RFLP	97	107	37	72	61	24	0.074	10
Wang et al., 2016	Chinese	NA	63.6/54.5	264/286	PCR-RFLP	107	121	36	126	117	43	0.071	12
						**IL-18 rs187238**							
	**East Asian**					**GG**	**GC**	**CC**	**GG**	**GC**	**CC**		
Pei et al., 2009 **^MI^**	Chinese	56.1 ± 10.2/55.1 ± 11.0	61.1/56.5	234/216	SSP-PCR	180	53	1	150	63	3	0.203	11
Liu et al., 2009 **^S/MVS^**	Chinese	60.3 ± 9.1/57.8 ± 7.9	74.7/54.5	241/145	LDR-PCR	195	46	0	99	44	2	0.101	10
Zhang et al., 2011 **^MI^**	Chinese	55.0 ± 9.0/56.0 ± 9.0	61.1/56.5	468/432	SSP-PCR	352	112	4	308	116	8	0.439	11
	**South Asian**												
Kumar et al., 2015	Indian	54.3 ± 12.1/52.0 ± 8.1	76.3/56.0	300/300	ASP-PCR	168	102	30	176	105	19	0.533	12
Khan et al., 2018	Indian	NA	78.0/68.0	200/260	ASP-PCR	37	145	18	38	215	7	<0.001	8
Ansari et al., 2017	Pakistani	42.0 ± 3.8/39.0 ± 7.8	96.8/96.1	340/310	TaqMan	217	116	7	183	108	19	0.568	12
	**Middle Easterner**												
Jabir et al., 2017/2021 **^&,S/MVS^**	Saudi	60.3± 9.2/47.9 ± 5.0	62.0/58.0	76/74	DNA sequencing	48	19	9	49	16	9	<0.001	7
Bazgir et al., 2018 **^S/MVS^**	Iranian	NA	NA	314/364	SSP-PCR	168	122	24	160	171	33	0.183	12
Hoseini et al., 2018 **^S/MVS^**	Iranian	59.4/56.7	56.0/51.0	100/100	ASP-PCR	57	39	4	48	46	6	0.242	12
Shayan et al., 2009 **^MI^**	Iranian	56.5/57.6	53.2/48.6	268/140	ASP-PCR	135	111	22	60	63	17	0.941	11
Borai et al., 2017	Egyptian	53.3± 8.3/NA	66.7/NA	60/50	SSP-PCR	27	27	6	12	28	10	0.389	10
	**Caucasian**												
Mitrokhin et al., 2018	Russian	71.0 ± 12.9/75.0 ± 7.4	55.7/28.4	176/112	TaqMan	86	76	14	52	48	12	0.853	11
Opstad et al., 2011	Norwegian	62.0/55.0	78.0/NA	995/204	TaqMan	532	394	69	108	82	14	0.768	14
Kariž et al., 2011 **^MI^**	Slovenian	61.0 ± 11.9/66.2 ± 9.8	66.9/46.0	169/326	TaqMan	90	71	8	162	141	23	0.301	10
Sierra Alique et al., 2005	Spanish	NA	69.0/NA	151/88	SSP-PCR	79	60	12	43	33	12	0.178	11
						**IL-18 rs1946518**							
	**East Asian**					**CC**	**CA**	**AA**	**CC**	**CA**	**AA**		
Pei et al., 2009 **^MI^**	Chinese	56.1 ± 10.2/55.1 ± 11.0	61.1/56.5	234/216	SSP-PCR	82	107	45	42	108	66	0.854	11
Ma et al., 2016	Chinese	NA	65.6/65.6	326/326	PCR-RFLP	90	128	108	43	158	125	0.530	12
Zhu et al., 2011	Chinese	54.7 ± 12.3/53.4 ± 11.3	53.9/53.3	141/240	SSP-PCR	47	71	23	51	123	66	0.653	10
Zhang et al., 2011 **^MI^**	Chinese	55.0 ± 9.0/56.0 ± 9.0	61.1/56.5	468/432	SSP-PCR	170	210	88	90	220	122	0.616	11
	**Middle Easterner**												
Jabir et al., 2017/2021 **^&,S/MVS^**	Saudi	60.3 ± 9.2/47.9 ± 5.0	62.0/58.0	74/74	DNA sequencing	65	0	9	59	2	13	<0.001	7
Bazgir et al., 2018	Iranian	NA	NA	314/364	SSP-PCR	109	153	52	136	178	50	0.494	12
Shayan et al., 2009 **^MI^**	Iranian	56.5/57.6	53.2/48.6	251/127	ASP-PCR	97	124	30	48	65	14	0.247	11
Borai et al., 2017	Egyptian	53.3 ± 8.3/NA	66/7/NA	60/50	SSP-PCR	12	39	9	4	42	4	<0.001	7
	**Caucasian**												
Mitrokhin et al., 2018	Russian	71.0 ± 12.9/75.0 ± 7.4	55.7/28.4	174/114	TaqMan	56	90	28	28	66	20	0.081	11
Opstad et al., 2011	Norwegian	62.0/55.0	78.0/NA	996/204	TaqMan	364	500	132	74	96	34	0.762	14
Kariž et al., 2011 **^MI^**	Slovenian	61.0 ± 11.9/66.2 ± 9.8	66.9/46.0	169/326	TaqMan	55	86	28	109	158	59	0.895	10
						**IL-18 rs1946519**							
						**GG**	**GT**	**TT**	**GG**	**GT**	**TT**		
Jabir et al., 2017/2021 **^&,S/MVS^**	Saudi	60.3 ± 9.2/47.9 ± 5.0	62.0/58.0	68/62	DNA sequencing	30	29	9	21	26	15	0.227	10
Ansari et al., 2017	Pakistani	42.0 ± 3.8/39.0 ± 7.8	96.8/96.1	340/310	TaqMan	194	108	38	152	115	43	0.007	9
Koch et al., 2011 **^MI^**	German	63.0 ± 12.5/60.3 ± 11.9	73.8/51.6	2136/1211	TaqMan	781	10211	334	406	590	215	0.979	15
						**IL-16 rs8034928**							
						**TT**	**TC**	**CC**	**TT**	**TC**	CC		
Huang et al., 2013	Chinese	62.1 ± 9.8/59.3 ± 10.4	80.5/67.5	651/428	PCR-RFLP	251	315	85	103	229	96	0.145	13
Tan et al., 2013	Chinese	63.1 ± 7.8/62.4 ± 8.5	66.2/54.2	260/281	PCR-MALDI-TOF MS	110	85	65	146	89	46	<0.001	9
Tong et al., 2013	Chinese	61.4 ± 8.7/60.6 ± 9.6	74.6/61.6	326/341	PCR-MALDI-TOF MS	167	106	53	193	123	25	0.382	12
						**IL-16 rs3848180**							
						**TT**	**TG**	**GG**	**TT**	**TG**	**GG**		
Huang et al., 2013	Chinese	62.1 ± 9.8/59.3 ± 10.4	80.5/67.5	651/428	PCR-RFLP	191	344	116	104	231	93	0.097	13
Tan et al., 2013	Chinese	63.1 ± 7.8/62.4 ± 8.5	66.2/54.2	260/281	PCR-MALDI-TOF MS	84	79	97	116	90	75	<0.001	9
Tong et al., 2013	Chinese	61.4 ± 8.7/60.6 ± 9.6	74.6/61.6	326/341	PCR-MALDI-TOF MS	105	131	90	110	141	90	0.002	9
						**IL-16 rs1131445**							
						**TT**	**TC**	**CC**	**TT**	**TC**	**CC**		
Huang et al., 2013	Chinese	62.1 ± 9.8/59.3 ± 10.4	80.5/67.5	651/428	PCR-RFLP	299	295	57	186	196	46	0.596	13
Tan et al., 2013	Chinese	63.1 ± 7.8/62.4 ± 8.5	66.2/54.2	260/281	PCR-MALDI-TOF MS	120	90	50	136	95	50	<0.001	9
Tong et al., 2013	Chinese	61.4 ± 8.7/60.6 ± 9.6	74.6/61.6	326/341	PCR-MALDI-TOF MS	157	112	57	171	116	54	<0.001	9
						**IL-16 rs4778889**							
						**CC**	**CT**	**TT**	**CC**	**CT**	**TT**		
Tong et al., 2013	Chinese	61.4 ± 8.7/60.6 ± 9.6	74.6/61.6	326/341	PCR-MALDI-TOF MS	170	106	50	201	107	33	0.002	9
Wu et al., 2011	Chinese	62.8 ± 11.6/61.6 ± 10.4	60.5/58.9	157/202	PCR-RFLP	7	55	95	10	75	117	0.647	11
Tan et al., 2013	Chinese	63.1 ± 7.8/62.4 ± 8.5	66.2/54.2	260/281	PCR-MALDI-TOF MS	139	88	33	151	91	39	<0.001	9
Chen et al., 2011	Chinese	57.2 ± 7.8/59.1 ± 11.6	81.0/66.2	300/397	PCR-RFLP	10	117	173	18	138	241	0.775	12
						**IL-16 rs11556218**							
						**GG**	**GT**	**TT**	**GG**	**GT**	**TT**		
Tong et al., 2013	Chinese	61.4 ± 8.7/60.6 ± 9.6	74.6/61.6	326/341	PCR-MALDI-TOF MS	223	69	34	249	80	12	0.088	12
Wu et al., 2011	Chinese	62.8 ± 11.6/61.6 ± 10.4	60.5/58.9	157/202	PCR-RFLP	4	92	61	8	87	107	0.057	11
Tan et al., 2013	Chinese	63.1 ± 7.8/62.4 ± 8.5	66.2/54.2	260/281	PCR-MALDI-TOF MS	175	64	21	191	62	28	<0.001	9
Chen-1 et al., 2011 **^#^**	Chinese	57.2 ± 7.8/59.1 ± 11.6	81.0/66.2	300/397	PCR-RFLP	23	235	42	16	149	232	0.187	12
Chen-2 et al., 2011 **^#^**	Chinese	57.2 ± 7.8/59.1 ± 11.6	81.0/66.2	424/332	PCR-RFLP	12	264	148	10	144	178	0.002	9
Silva et al., 2020	Brazilian	NA/48.0 ± 6.3	NA/875.4	250/220	PCR-RFLP	5	58	187	1	78	141	0.004	8

HWE: Hardy–Weinberg Equilibrium in control groups; NA: Not applicable; PCR-RFLP: Polymerase chain reaction-restriction fragment length polymorphism; TP-ARMS-PCR: Tetra-primer-amplification refractory mutation system-PCR; TaqMan: PCR allelic discrimination with TaqMan probes; SSP-PCR: Sequence specific primer-PCR; LDR-PCR: Ligase detection reaction-PCR; ASP-PCR: Allele specific primer-PCR; PCR-MALDI-TOF MS: PCR and matrix-assisted laser desorption/ionization time-of-flight mass spectrometry. ^MI^ Author, Year: Coronary artery disease (CAD) Patients with a manifestation of myocardial infarction included in the study; ^ACS^ Author, Year: CAD Patients with a manifestation of acute coronary syndrome included in the study; ^S/MVS^ Author, Year: CAD Patients with a manifestation of single/multiple vessel stenosis included in the study. ^§^ Quality Score range: 0–15; higher scores represent better quality. * Author, Year: Two independent cohorts for investigation of IL-8 SNPs (rs4073 and rs2227306) included in one publication; ^&^ Author, Year: Overlapping participants existed in two publications, one of which with the manifestation study emphasis only; ^#^ Author, Year: Two independent cohorts for investigation of IL-16 rs11556218 polymorphism included in one publication.

**Table 2:** Pooled meta-analysis for associations between IL-8 SNPs and the risk of CAD.

SNPs (Alleles) ^#^	Overall or Subgroup (No. of Cohort)	Sample Size Case/Control	Allelic Model			Homozygous Model			Heterozygous Model			Dominant Model		
			EM	OR (95% CI)	*p*	EM	OR (95% CI)	*p*	EM	OR (95% CI)	*p*	EM	OR (95% CI)	*p*
rs4073 (T > A)	Overall (15)	5126/5346	R	1.26 (1.09, 1.45)	0.002	R	1.50 (1.15, 1.95)	0.003	R	1.25 (1.05, 1.49)	0.01	R	1.34 (1.10, 1.64)	0.004
	Ethnicity													
	Asian (10)	3392/3384	R	1.46 (1.27, 1.66)	<0.001	R	1.96 (1.57, 2.45)	<0.001	F	1.47 (1.32, 1.65)	<0.001	R	1.65 (1.38, 1.97)	<0.001
	Chinese (8)	2842/2834	F	1.34 (1.24, 1.44)	<0.001	F	1.72 (1.48, 2.00)	<0.001	F	1.38 (1.22, 1.55)	<0.001	F	1.47 (1.32, 1.65)	<0.001
	Non-Chinese (2)	550/550	R	2.74 (1.01, 7.47)	<0.05	R	4.25 (1.52, 11.91)	0.006	F	2.37 (1.72, 3.27)	<0.001	F	2.74 (2.04, 3.66)	<0.001
	Caucasian (5)	1734/1962	F	0.91 (0.83, 1.00)	0.06	F	0.82 (0.68, 0.99)	0.04	F	0.86 (0.73, 1.01)	0.06	F	0.85 (0.73, 0.99)	0.04
	Sample size													
	Large size (3)	2042/2171	R	1.27 (0.89, 1.81)	0.18	R	1.59 (0.78, 3.23)	0.20	R	1.34 (0.78, 2.31)	0.29	R	1.42 (0.79, 2.54)	0.24
	Small size (12)	3084/3175	R	1.25 (1.06, 1.48)	0.007	R	1.47 (1.10, 1.95)	0.009	F	1.25 (1.11, 1.40)	<0.001	R	1.32 (1.07, 1.62)	0.009
	Quality score													
	≥10 (12)	4341/4544	R	1.21 (1.03, 1.41)	0.02	R	1.42 (1.05, 1.91)	0.02	R	1.24 (1.01, 1.53)	0.04	R	1.29 (1.03, 1.63)	0.03
	<10 (3)	785/802	R	1.67 (1.00, 2.79)	0.05	R	2.00 (0.97, 4.11)	0.06	F	1.25 (0.98, 1.58)	0.07	R	1.63 (0.97, 2.72)	0.06
	Clinical manifestation													
	ACS (5)	1480/1543	R	1.08 (0.88, 1.34)	0.46	R	1.10 (0.69, 1.74)	0.69	F	1.22 (1.04, 1.43)	0.01	R	1.18 (0.93, 1.51)	0.17
	Non-ACS (3)	290/547	R	1.16 (0.77, 1.76)	0.48	R	1.36 (0.56, 3.33)	0.50	F	1.26 (0.90, 1.78)	0.18	F	1.34 (0.97, 1.85)	0.08
rs2227306 (C > T)	Overall (5)	2642/2853	F	0.99 (0.91, 1.07)	0.75	F	0.97 (0.83, 1.14)	0.75	F	0.98 (0.87, 1.10)	0.75	F	0.98 (0.88, 1.10)	0.74
	Clinical manifestation													
	ACS (3)	1156/1153	F	1.02 (0.91, 1.15)	0.72	F	1.06 (0.82, 1.36)	0.66	F	0.99 (0.83, 1.19)	0.95	F	1.01 (0.85, 1.19)	0.91

EM: Effect model; R: Random-effect model; F: Fixed-effect model. MI: Myocardial infarction; ACS: Acute coronary syndrome. ^#^: Major and minor alleles in the meta-population.

**Table 3:** Pooled meta-analysis for associations between IL-18 SNPs and the risk of CAD.

SNPs (Alleles) ^#^	Overall or Subgroup (No. of Cohort)	Sample Size Case/Control	Allelic Model			Homozygous Model			Heterozygous Model			Dominant Model		
			EM	OR (95% CI)	*p*	EM	OR (95% CI)	*p*	EM	OR (95% CI)	*p*	EM	OR (95% CI)	*p*
**rs187238** **(G > C)**	**Overall (15)**	4092/3121	F	0.85 (0.79, 0.93)	<0.001	F	0.75 (0.61, 0.94)	0.01	F	0.82 (0.74, 0.92)	<0.001	F	0.81 (0.73, 0.90)	<0.001
	Ethnicity													
	East Asian (3)	943/793	F	0.72 (0.59, 0.88)	0.001	F	0.33 (0.12, 0.89)	0.03	F	0.73 (0.59, 0.91)	0.005	F	0.71 (0.57, 0.88)	0.002
	South Asian (3)	840/870	R	0.99 (0.77, 1.27)	0.91	R	1.11 (0.34, 3.61)	0.86	F	0.90 (0.73, 1.12)	0.35	F	0.91 (0.74, 1.12)	0.39
	Middle Easterner (5)	818/728	F	0.76 (0.65, 0.89)	<0.001	F	0.63 (0.43, 0.90)	0.01	F	0.72 (0.58, 0.89)	0.003	F	0.71 (0.58, 0.87)	<0.001
	Caucasian (4)	1491/730	F	0.91 (0.78, 1.06)	0.21	F	0.75 (0.52, 1.10)	0.14	F	0.95 (0.78, 1.17)	0.65	F	0.92 (0.76, 1.12)	0.40
	Sample size													
	Large size (1)	995/204	NA	0.99 (0.78, 1.26)	0.92	NA	1.00 (0.54, 1.84)	1.00	NA	0.98 (0.71, 1.34)	0.88	NA	0.98 (0.72, 1.32)	0.89
	Small size (14)	3097/2917	F	0.84 (0.77, 0.91)	<0.001	F	0.72 (0.57, 0.91)	0.007	F	0.81 (0.72, 0.90)	<0.001	F	0.79 (0.71, 0.88)	<0.001
	Quality score													
	≥10 (13)	3816/2787	F	0.83 (0.76, 0.90)	<0.001	F	0.69 (0.55, 0.86)	0.001	F	0.83 (0.74, 0.92)	0.001	F	0.81 (0.72, 0.90)	<0.001
	<10 (2)	276/334	F	1.06 (0.83, 1.34)	0.65	F	1.68 (0.84, 3.35)	0.14	F	0.82 (0.54, 1.24)	0.35	F	0.87 (0.59, 1.30)	0.51
	Clinical manifestation													
	MI (4)	1007/1114	F	0.81 (0.69, 0.95)	0.009	F	0.55 (0.33, 0.93)	0.02	F	0.84 (0.69, 1.01)	0.07	F	0.80 (0.67, 0.97)	0.02
	SVS (4)	282/658	R	1.06 (0.68, 1.65)	0.79	F	0.74 (0.38,1.42)	0.36	R	1.37 (0.67,2.78)	0.39	R	1.28 (0.64,2.55)	0.48
	MVS (4)	447/658	R	0.54 (0.35, 0.83)	0.005	F	0.64 (0.38, 1.09)	0.10	R	0.45 (0.28, 0.72)	<0.001	R	0.45 (0.28, 0.73)	0.001
rs1946518 (C > A)	Overall (11)	3207/2473	R	0.79 (0.67, 0.92)	0.003	R	0.62 (0.45, 0.85)	0.003	R	0.69 (0.53, 0.90)	0.006	R	0.67 (0.51, 0.87)	0.003
	Ethnicity													
	East Asian (4)	1169/1214	F	0.62 (0.55, 0.70)	<0.001	F	0.38 (0.30, 0.48)	<0.001	F	0.49 (0.40, 0.60)	<0.001	F	0.45 (0.38, 0.55)	<0.001
	Middle Easterner (4)	699/615	F	1.02 (0.87, 1.20)	0.81	F	1.08 (0.76, 1.54)	0.65	F	0.94 (0.73, 1.22)	0.67	F	0.96 (0.76, 1.22)	0.76
	Caucasian (3)	1339/644	F	0.92 (0.80, 1.08)	0.31	F	0.82 (0.60, 1.12)	0.21	F	0.98 (0.78, 1.24)	0.88	F	0.94 (0.75, 1.18)	0.59
	Sample size													
	Large size (1)	996/204	NA	0.93 (0.74, 1.15)	0.49	NA	0.79 (0.50, 1.24)	0.31	NA	1.06 (0.76, 1.48)	0.74	NA	0.99 (0.72, 1.35)	0.94
	Small size (10)	2211/2269	R	0.77 (0.65, 0.92)	0.004	R	0.60 (0.43, 0.85)	0.004	R	0.66 (0.50, 0.86)	0.003	R	0.63 (0.48, 0.84)	0.002
	Quality score													
	≥10 (9)	3073/2349	R	0.79 (0.67, 0.94)	0.008	R	0.62 (0.44, 0.87)	0.006	R	0.72 (0.55, 0.94)	0.02	R	0.69 (0.52, 0.92)	0.01
	<10 (2)	134/124	F	0.76 (0.51, 1.14)	0.19	F	0.66 (0.29, 1.46)	0.30	F	0.28 (0.09, 0.87)	0.03	F	0.46 (0.22, 0.94)	0.03
	Clinical manifestation													
	MI (4)	1001/1101	R	0.75 (0.57, 1.00)	0.05	R	0.59 (0.33, 1.03)	0.06	R	0.68 (0.46, 1.00)	0.05	R	0.65 (0.42, 1.01)	0.051
rs1946519 (G > T)	Overall (3)	2544/1583	F	0.87 (0.79, 0.95)	0.003	F	0.77 (0.64, 0.93)	0.007	F	0.87 (0.75, 1.00)	0.04	F	0.84 (0.73, 0.95)	0.008

EM: Effect model; R: Random-effect model; F: Fixed-effect model; NA: Not applicable. MI: Myocardial infarction; SVS: single vessel stenosis; MVS: multiple vessel stenosis. ^#^: Major and minor allele in the meta-population.

**Table 4:** Pooled meta-analysis for associations between IL-16 SNPs and the risk of CAD.

SNPs (Alleles) ^#^	Overall or Subgroup (No. of Cohort)	Sample Size Case/Control	Allelic Model			Homozygous Model			Heterozygous Model			Dominant Model		
			EM	OR (95% CI)	*p*	EM	OR (95% CI)	*p*	EM	OR (95% CI)	*p*	EM	OR (95% CI)	*p*
rs8034928 (T > C)	Overall (3)	1237/1050	R	1.08 (0.59, 2.00)	0.80	R	1.18 (0.34, 4.09)	0.80	R	0.88 (0.54, 1.43)	0.61	R	0.97 (0.49, 1.91)	0.93
rs3848180 (T > G)	Overall (3)	1237/1050	R	1.07 (0.78, 1.48)	0.67	R	1.08 (0.62, 1.86)	0.79	F	0.94 (0.77, 1.15)	0.54	R	1.03 (0.72, 1.49)	0.87
rs1131445 (T > C)	Overall (3)	1237/1050	F	1.00 (0.88, 1.13)	0.98	F	1.00 (0.77, 1.28)	0.98	F	1.00 (0.83, 1.19)	0.98	F	1.00 (0.85, 1.18)	1.00
rs4778889 (T > C)	Overall (4)	1043/1221	F	0.91 (0.80, 1.05)	0.19	F	0.77 (0.57, 1.05)	0.10	F	1.00 (0.81, 1.23)	0.99	F	0.95 (0.78, 1.16)	0.62
	Quality Score													
	≥10 (2)	457/599	F	1.00 (0.82, 1.23)	0.96	F	0.81 (0.43, 1.51)	0.50	F	1.08 (0.84, 1.39)	0.56	F	1.05 (0.82, 1.34)	0.71
	<10 (2)	586/622	R	0.86 (0.63, 1.19)	0.37	R	0.77 (0.40, 1.49)	0.44	R	0.86 (0.50, 1.48)	0.58	R	0.81 (0.43, 1.49)	0.49
rs11556218 (T > G)	Overall (6)	1717/1773	R	1.22 (0.76, 1.95)	0.24	R	1.48 (0.52, 4.18)	0.46	R	1.46 (0.60, 3.51)	0.40	R	1.44 (0.61, 3.39)	0.41
	Quality Score													
	≥10 (3)	783/940	R	1.41 (0.57, 3.50)	0.46	R	1.31 (0.14, 1.91)	0.81	R	1.74 (0.32, 9.43)	0.52	R	1.72 (0.31, 9.51)	0.53
	<10 (3)	934/833	R	1.07 (0.68, 1.67)	0.78	F	1.39 (0.86, 2.23)	0.18	R	1.20 (0.47, 3.05)	0.71	R	1.18 (0.51, 2.77)	0.70

EM: Effect model; R: Random-effect model; F: Fixed-effect model. ^#^: Major and minor alleles in the meta-population.

**Table 5:** Egger’s test for funnel plot asymmetries.

Gene SNPs (Alleles) ^#^	*p*			
	Allelic Model	Homozygous Model	Heterozygous Model	Dominant Model
IL-8 rs4073 (T > A)	0.251	0.399	0.609	0.437
IL-18 rs187238 (G > C)	0.092	0.087	0.336	0.192
IL-18 rs1946518 (C > A)	0.868	0.536	0.307	0.437
IL-16 rs11556218 (T > G)	0.127	0.823	0.401	0.365

**^#^**: Major and minor alleles in the meta-population.

## Data Availability

The original contributions presented in this study are included in the article/Supplementary Material. Further inquiries can be directed to the corresponding author(s).

## Supplementary Materials

The following supporting information can be downloaded at https://www.mdpi.com/article/10.3390/biom14121631/s1, Table S1. Computation-based prediction of functional effects of the SNPs. Various bioinformatics tools were utilized to predict the functional effects of ten vari-ants analyzed in this study [113,114,115,116,117].
