# Supplementary material for: Cytokine Gene Variants as Predisposing Factors for the Development and Progression of Coronary Artery Disease: A Systematic Review
Source: Biomolecules. 2024 Dec 19;14(12):1631. doi: 10.3390/biom14121631 (PMC11726869; doi:10.3390/biom14121631)
Supplement: Supplementary file 1 [file biomolecules-14-01631-s001.zip › biomolecules-3344664-supplementary.pdf]

Supplemental material

Method : bioinformatics analysis of functional effect prediction of variants

Two web databases, RegulomeDB[1-2] (http://regulomedb.org) and HaploReg v4.2[3] (https://www.broadinstitute.org/mammals/haploreg/haploreg.php) were applied to predict the potential regulatory role of non-coding SNPs. Concurrently, prediction of functional impact of the missense variant was performed by using two additional web-based softwares, PolyPhen-2[4] (http://genetics.bwh.harvard.edu/pph2/) and Mutation Taster[5] (https://www.mutationtaster.org/).

Results: computational analysis of predicted functional effects of variants

To analyze the probability of investigated variants in this study to be of functional significance, four online tools were employed finally. According to Supplemental Table 1, RegulomeDB scores belonging to category 1 were assigned to eight out of nine non-coding SNPs, providing strong evidence for regulatory function. It offered further clues regarding regulatory potential of these variants in combination with usage of HaploReg v4.2. The 9 non-coding SNPs were associated with histone modification markers, as well as with a DNase I hypersensitive site (except rs1946518). Not surprisingly, rs2227306 was the expression quantitative trait loci (eQTL) affecting expression of IL-8. This SNP was also eQTLs for CXCL6 and PF4V1 with another functional variant rs4073. The latter mapped to eleven proteins binding sites and mediated the binding affinity of STAT. Rs2227306 fell within over 50 different protein binding sites which displaying overlaps with that of rs4073 fell (i.e. CEBPA, CEBPB, POLR2A, JUN, NR3C1, CEBPG, TCF12 and ATF3), and changed a serial of binding motifs (CEBPB, Sox, Foxp1, HNF1 and Pou3f2). All the three functional proxies in the IL-18 gene region, including intergenic polymorphisms rs187238, rs1946518 and rs1946519 were eQTLs for 8 proteins (IL-18, BCO2, PTS etc). The variant rs187238 situated within the binding sites of 33 proteins and altered Foxd3 binding motif, while rs1946518 lay within binding site of SP11 and disrupted 15 transcription factors binding motifs. Three functional SNPs rs8034928, rs3848180

and rs4778889 in intronic locations had indications for different proteins binding of SP1, REST, and so forth. Similar to the three SNPs, rs1131445 were eQTLs for IL-16 and STARD5. The intronic variant rs3848180 exhibited more evidence of regulatory potential with a score of 1b: situated within the binding sites of 14 proteins and changed the motifs of transcription factor Nkx2 and HMX family members. Neither the four functional proxies on the IL-16 locus nor the functional proxy on the IL-8 gene region were predicted as risk factor based on Mutation Taster. Predictions were not available for other non-coding SNPs.

Locating in a non-synonymous coding area of IL-16, the variant rs11556218 presented within protein binding site of POLR2A (a subunit of DNA-directed RNA polymerase II which is involved in mRNA processing and chromatin remodelling) and GATA3 (a transcription factor which exerted multiple function in immune system). It also alters SP1, SP2 and YY1 motifs. Prediction analysis of functional effects suggested that this missense SNP is largely likely to be deleterious to protein function, thereby supporting an evidence of the causal variant and candidate risk loci for CAD (e.g. PolyPhen-2 score $\geqslant$0.957 with a prediction as “probably damaging”).

References

1. Dong S, Zhao N, et al. (2022). Annotating and prioritizing human non-coding variants with RegulomeDB. In bioRxiv.

2. Boyle AP, Hong EL, et al (2012). Annotation of functional variation in personal genomes using RegulomeDB. Genome research 22, 1790–1797.

3. Ward LD. and Kellis M (2016). HaploReg v4: systematic mining of putative causal variants, cell types, regulators and target genes for human complex traits and disease. Nucleic acids research 44, D877–D881.

4. Adzhubei IA et al (2010). A method and server for predicting damaging missense mutations. Nat Methods, 7(4): 248-249.

5. Schwarz JM, Cooper DN, et al (2014). MutationTaster2: mutation prediction for the deep-sequencing age. Nat Methods, 11(4): 361-362.

Supplementary Table S1. Computation based prediction of functional effects of the SNPs

Gene	dbSNP ID	Allele	Chromosome Location (GRCh38.p7)	SNP Location	PHM /EHM /DNase	Regulome DB Rank (Category)#	HaploReg v4.1 /RegulomeDB Protein bound	HaploReg v4.1 /RegulomeDB Motif altered	HaploReg v4.1 /RegulomeDB eQTL	Polyphen 2_HDIV Score	Mutation Taster Score
IL-8	rs4073	A/T	4:73740307	Intergenic<br>198 bp<br>5' of IL-8	+<br>/+<br>/+	1f<br>(1)	AP2ALPHA,<br>AP2GAMMA,<br>JUND*<br>/ 51 additional binding proteins	CEBPB, Sox, Foxp1,<br>HNF1, Pou3f2<br>/ —	—<br>/ CXCL6, PF4V1	—	—
IL-8	rs2227306	C/T	4:73741338	Intron 1	+<br>/+<br>/+	1f<br>(1)	CEBPB*<br>/ 10 additional binding proteins	STAT<br>/ —	CXCL6*, IL-8*,<br>PF4V1*	—	0.99999<br>(Probably harmless)
Il-18	rs187238	C/G	11:112164265	Intergenic<br>147 bp<br>5' of IL-18	+<br>/+<br>/+	1f<br>(1)	TBP, POL2<br>/ 31 additional binding proteins	Foxd3<br>/ —	RP11-356J5.5,<br>RPS12P21,<br>BCO2*, IL-18*,<br>MRPS36P4*,<br>RP11-356J5.12*<br>/ PTS, KCTD9P4<br>RP11-65M17.3	—	—
Il-18	rs1946518	T/G	11:112164735	Intergenic<br>617 bp<br>5' of IL-18	+<br>/+<br>/—	1f<br>(1)	—<br>/ SP11	CEBPA, DMRT3,<br>DMRT7, Hoxa10,<br>Dbx1, HNF1, Ncx,<br>ATF2, Hlx1, Mef2,<br>TATA, Nkx6, Zfp187,<br>Arid3a*<br>/ PHOX2B	RP11-356J5.5,<br>RPS12P21<br>BCO2*, IL-18*,<br>MRPS36P4*, PTS*<br>RP11-356J5.12*<br>/ TEX12, KCTD9P4	—	—
Il-18	rs1946519	A/C	11:112164784	Intergenic<br>666 bp<br>5' of IL-18	+<br>/+<br>/+	7<br>(5)	—<br>/ —	RXRA<br>/ —	RP11-356J5.5,<br>RPS12P21,<br>BCO2*, IL-18*,<br>MRPS36P4*, PTS*<br>RP11-356J5.12*<br>/ TEX12, KCTD9P4	—	—
IL-16	rs8034928	T/C	15:81301782	Intron 15	+<br>/+<br>/+	1f<br>(1)	NFKX, POL2,<br>FOXA1*<br>/ 23 additional binding proteins	Duxl, FXR, Gfl1b,<br>LXR, SF1<br>/ ESRRA	IL-16, STARD5<br>/ RP11-761I4.4	—	0.99999<br>(Probably harmless)
IL-16	rs3848180	T/G	15:81304249	Intron 16	+<br>/+<br>/+	1b<br>(1)	—<br>/ 14 additional binding proteins	Hmx, Nkx2<br>/ HMX1, HMX2, HMX3	IL-16<br>/ STARD5,<br>RP11-761I4.4	—	0.99999<br>(Probably harmless)
IL-16	rs1131445	T/C	15:81309441	3'-UTR	+<br>/+<br>/+	1f<br>(1)	POL2<br>/ POLR2A, PML	—<br>/ —	IL-16, STARD5*<br>/ RP11-761I4.4	—	0.99999<br>(Probably harmless)
IL-16	rs4778889	T/C	15:81296654	Intron 12	+<br>/+<br>/+	1f<br>(1)	—<br>/ 19 additional binding proteins	Sox, TAL1<br>/ —	IL-16*<br>/ STARD5	—	0.99994<br>(Probably harmless)
IL-16	rs11556218	T/G	15:81305928	Exon 17<br>missense	—<br>/+<br>/+	4<br>(4)	—<br>/ POLR2A,<br>GATA3	SP1, SP2, YY1<br>/ —	—<br>/ —	0.987<br>Probably damaging	0.00959<br>(Probably deleterious)

PHM:promoter histone marks; EHM: enhancer histone marks; eQTL: expression quantitative trait loci; #Category: Capability of affecting protein binding and expression of a gene target, ranged from 1 (most likely)-5 (lest likely); *:The repeated sites in both database.
